# How expensive are post-traumatic stress disorders? Estimating incremental health care and economic costs on anonymised claims data

**DOI:** 10.1007/s10198-020-01184-x

**Published:** 2020-05-26

**Authors:** Tim Bothe, Josephine Jacob, Christoph Kröger, Jochen Walker

**Affiliations:** 1 Department of Psychology, Technical University of Brunswick, Humboldtstraße 33, 38106 Brunswick, Germany; 2 InGef – Institute for Applied Health Research Berlin, Spittelmarkt 12, 10117 Berlin, Germany; 3 Department of Psychology, University of Hildesheim, Universitätsplatz 1, 31141 Hildesheim, Germany

**Keywords:** Post-traumatic stress disorders, Health care costs, Claims data, Cost-analysis, Cost-comparison, I18 (Public Health), I19 (Health – Other)

## Abstract

**Background:**

Post-traumatic stress disorders (PTSD) pose a high burden for individuals and societies. Although prevalence rates are rather low, high co-occurrence rates and overall impairments cause deleterious suffering and significant costs. Still, no long-term data on costs and trends in cost developments are available.

**Methods:**

Claims data from a German research database were analysed regarding direct and indirect costs occurring for individuals with incident diagnoses of PTSD. Results were compared to non-exposed average insurants matched on age and gender. Costs were analysed over a 5-year period from 2 years preceding until 3 years following an incident diagnosis of PTSD.

**Results:**

Overall costs for PTSD account for approximately 43,000 EUR per individual, which is three times higher than costs for non-exposed controls. Of these costs, 59% are caused by mental disorders, 18% specifically by PTSD. In the control group, costs for mental disorders account for 19% of total costs. Costs increase by 142% in the year after an incident diagnosis of PTSD but return to the initial level 2 years later. Still, costs are at least twice as high in every year as in those for the comparison group.

**Conclusions:**

Individuals with PTSD seem to suffer from far more impairments in their general health conditions and incur many more costs than average insurants. Most of these seem to be caused by co-occurring mental disorders and show their maximum in the index year. Nevertheless, as costs decrease to their initial level, treatments seem to have counterbalanced the impairments due to PTSD. Thus, treatments for PTSD can be considered as beneficial and their cost-effectiveness should be further investigated.

## Introduction

Post-traumatic stress disorders (PTSD) are a critical public health issue with significant impacts on affected individuals, health care systems, and societies. Despite comparatively low prevalence rates of 1.1–2.9% in the European population [1], the disability burden and occurring societal costs associated with PTSD are considered to be high [2]. Individuals suffer from PTSD symptoms for 6 years on average [3] and show a higher risk of suicide [4, 5]. Co-occurrence rates for other mental disorders are estimated at 50–100% [6]. In particular, depressive, anxiety, and substance use disorders are considered to be the most prevalent co-occurring mental disorders [4]. Furthermore, individuals with PTSD suffer from high impairments in their overall health condition and co-occurring physical disorders [7, 8]. Common impairments are increased rates of cardiovascular, pulmonary, and autoimmune diseases [9–11]. PTSD often causes incapacity to work and early retirements [12, 13], significant losses in quality of life, and increased utilisations of health care and other social services [2].

In general, mental disorders pose a serious burden to the European population. The overall 12-month prevalence rate of having any mental disorder is estimated at 25.7–38.2%, and their impact on global health impairments continues to increase [1]. Mental disorders cause steep direct (e.g., in- and outpatient treatments, medication) as well as indirect costs (e.g., sickness benefit payments, lost productivity, disability-adjusted life years) [14]. Despite immersive expenses, cost–benefit analyses estimate positive returns of investment for psychotherapeutic treatments specific to several mental disorders [15–19]. Still, not all individuals in need receive adequate treatments or get treated at all [1, 20, 21]. Thus, providing arguments for enabling improvements in health care for mental disorders is necessary [22]. Nevertheless, there is no subsequent clarity about the amount of monetary losses that arise due to mental disorders, and the potential for savings if individuals are treated. Without data to gain a better knowledge of the resulting costs, and to support population-based cost–benefit approaches, the estimates and hence the arguments for providing better health care for mental disorders are rather vague.

For PTSD, little is known about the costs that come along with the disorder’s impairments. As to our knowledge, only a few studies have investigated health care costs due to PTSD. Based on US-American private insurance and Medicaid data, one study estimated direct health care costs of 10,960–18,753 USD per patient per year [23]. A bottom-up study from Northern Ireland on interview data estimated total direct and indirect costs of 317,431,860 EUR for 74,935 affected individuals in the whole national population, which accounts for 4236 EUR for each individual with PTSD [24]. However, comparability of these findings and transferability to other populations and health care systems in other countries are limited due to different estimation approaches.

Thus, this study sought to add evidence regarding the quantity of monetary losses due to PTSD and to compare these costs to those incurred by non-exposed average insurants using a large database of anonymised German statutory health care claims data from the nationwide InGef research database. Data was analysed via all the available outcome measures of direct and indirect costs to estimate incremental costs and potential savings. Trends for cost developments were described to gain a broader view of PTSD and its course of illness before and after the onset of the disorder. Concluding the empirical evidence on the high individual and health burden associated with PTSD, we hypothesised that costs for individuals with PTSD exceed the costs resulting in non-exposed typical insurants after an incident diagnosis.

## Methods

### Data source

The study was based on anonymised claims data from approximately 70 nationwide statutory health insurance (SHI) providers and more than 8 million individuals included in the InGef research database. In addition to demographic data, the database contains information on dispensed drug prescriptions, outpatient and inpatient services, incapacities to work, and diagnoses. In 2013, the database showed good accordance with the overall German population regarding measures of morbidity, mortality, and drug use [25]. Information about diagnoses and drug prescriptions is available in accordance with the International Statistical Classification of Diseases and Related Health Problems, 10th Revision, German Modification (ICD-10-GM) [26], and the anatomical therapeutic chemical (ATC) code. Results are reported following the Consensus German Reporting Standard for Secondary Data Analyses (STROSA 2) [27].

### Study design and population

#### Selection of the PTSD-group

We conducted a retrospective cohort study of individuals with newly diagnosed PTSD, including data from 2010 until 2017. In a first step, individuals with a prevalent PTSD (ICD-10-GM F43.1) between 2012 and 2014 were identified by the following criteria: individuals had to have either (a) one or more inpatient diagnoses coded as ‘main diagnosis’, (b) two or more outpatient diagnoses stated as ‘secure diagnosis’, or (c) at least one ‘secure’ outpatient diagnosis by a specialist for mental disorders, e.g., a psychotherapist, a psychiatrist, or a neurologist. Incidence was defined as not having had a PTSD diagnosis, according to the abovementioned criteria, 2 years prior to the first PTSD diagnosis (index date) between 2012 and 2014. Only individuals who were fully observable in the database 2 years prior to the first PTSD diagnosis and in the 3 years following their index date or until their death were included. In addition, only individuals aged 18–65 at index date were included in the study population. Individuals were excluded from further analysis if they suffered from (a) organic, including symptomatic, mental disorders, (b) schizophrenia, schizotypal and delusional disorders, or (c) mental retardation (ICD-10-GM groups F0, F2, and F7) in the period 2 years prior to or in the quarter of their index date.

#### Selection of a non-exposed control group

To estimate the incremental costs that arise due to PTSD, a no-PTSD affected control group (No-PTSD CG) was identified. The same inclusion and exclusion criteria as for the PTSD-group were applied with the exception of the PTSD diagnosis. We assigned random index dates between 2012 and 2014 to all individuals that had no PTSD diagnosis. Only individuals who were fully observable 2 years prior to the assigned random index date and 3 years after their index date or until their death and who were aged 18–65 were included. Additionally, individuals were excluded if for a F0, F2, or F7 diagnosis in the 2 years preceding or in the quarter of their index diagnosis had been documented. A 1:4 matching by age and gender of individuals with PTSD and non-exposed individuals was conducted, and the resulting sample was used for further analysis.

### Baseline characteristics

Sociodemographic data such as gender, age, and insurance status were analysed. Comorbidities in the 2 years prior to the index date according to the ICD-10-GM chapters were assessed to gain a broader view of the overall health status of the study populations. Furthermore, Charlson comorbidity indices (CCI) [28] were determined to estimate individuals’ comorbidity, and, thus, their health status.

### Outcomes

#### Total health care costs

As a primary outcome, the overall amount of costs resulting from health care service utilisation and indirect costs for the German welfare state were analysed. Included were filed medication prescriptions, inpatient and outpatient treatments, sickness benefit payments, and costs due to losses of gross value per day absent. For the latter, average amounts of costs per day absent for every year from 2010 until 2017 as reported by the Federal Institute for Occupational Safety and Health [29] were used for calculations and multiplied with the actual number of days spent incapable to work during the respective analysis period. Costs are reported in total and for every outcome, respectively, and in total as well as yearly for the period 2 years prior to the index date, the year after an index diagnosis, and for the following 2 years. Results from both groups were compared to investigate differences in amounts of costs in the year after the onset of PTSD and in the preceding and following 2 years.

#### Costs resulting from mental disorder-specific health care services

In the second step, data were analysed for all costs due to mental disorders. Only services which were billed due to a documented mental disorder (ICD-10 GM chapter F) within the same hospital case or ambulatory treatment case were included in the cost analysis, as well as sickness benefit payments and reports of incapacities to work. For drug therapies, all prescriptions of ATC-codes beginning with ‘N05’ (psycholeptics) or ‘N06’ (psychoanaleptics) were considered as mental disorder-specific prescriptions in the calculation of mental disorder-related health care costs. Again, costs were compared between groups to analyse differences in costs caused by mental disorders as an estimate for other co-occurring disorders in individuals’ mental health status.

#### Costs resulting from non-mental disorder-specific health care services

Vice versa, costs that could be identified as not caused by mental disorders were reported using the same method. All services and treatments in which no mental disorder was documented were included in this sub-analysis. Analysis followed the same procedure as for the other outcomes, and costs were compared between groups.

#### PTSD-specific health care service utilisation

Furthermore, the PTSD-group was analysed for any cost factors due to PTSD after its onset to gain an overview of trends in disorder-specific cost developments for an incident PTSD. Following the same procedure as abovementioned, all services containing a diagnosis of PTSD (ICD-10-GM F43.1) were included in this analysis. As drug therapies cannot be identified as being prescribed explicitly for PTSD or other mental disorders, no PTSD-specific drug costs were estimated.

### Statistical analysis

Data were extracted, aggregated, and analysed using R version 3.5.0. Analyses were conducted separately for every year during the 2 years preceding an index diagnosis, for the year following an index diagnosis, and for the 2 years after, as well as for the whole 5-year observation period. All descriptive and statistical analyses were conducted regarding total sample sizes, respectively. Differences in totals, i.e., for insurance status and for monetary values, can occur due to multiple data for a respective analysis period, or due to accumulation of overlapping intervals for continuous services.

In accordance with current research trends [30, 31], we focused on descriptive analyses and interpretation of confidence intervals (CI) and effect sizes. These are reported as Cramer’s *V* or Cohen’s *d*. For completeness, we also reported Chi-square tests for categorical variables and Student’s *t* tests with Bonferroni correction for continuous variables. Considering the large sample sizes, differences were considered significant at a level of *p* < 0.001.

## Results

### Group characteristics and comorbidities

**Fig. 1:**
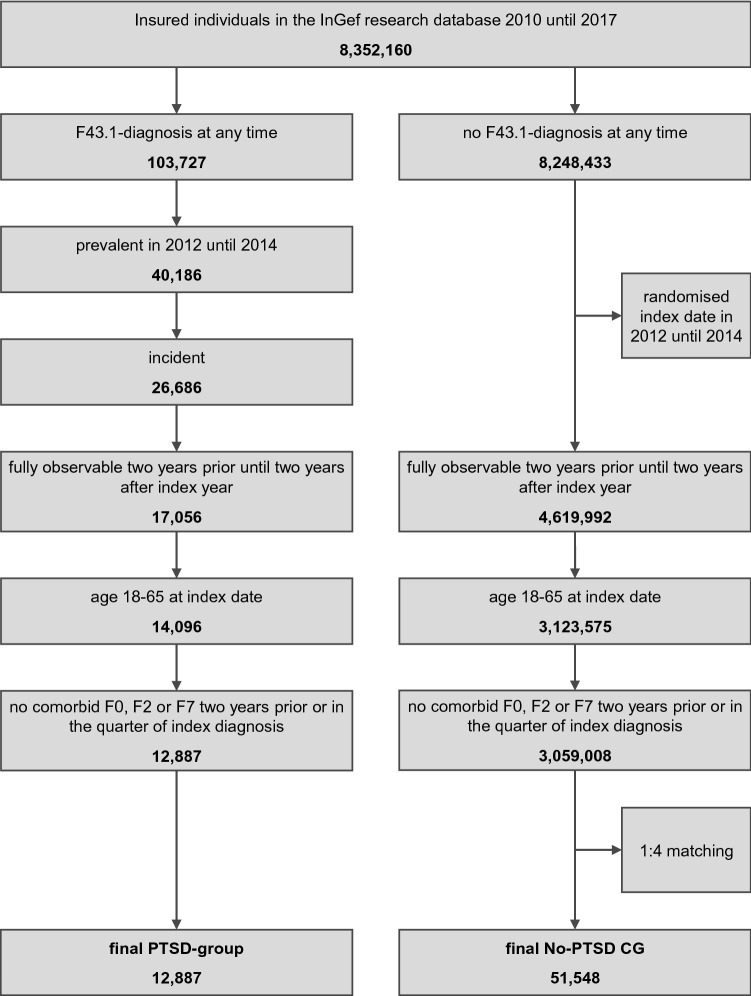
Flowchart for sample identification process

Between 2010 and 2017, anonymised data for approximately 8.4 million individuals were available in the research database. Of these, 103,727 received a diagnosis of PTSD (F43.1) at any time, while 8,248,433 did not. In the PTSD-cohort, 40,186 were identified as prevalent, and 26,686 as an incident between 2012 and 2014. Of these, 17,056 were fully observable in the whole analysis period, and 14,096 were aged 18 to 65. A total of 1,209 individuals were excluded due to a diagnosis of F0, F2, or F7 within 2 years prior to or in the quarter of their index date, which resulted in a final study sample of 12,887 individuals with PTSD. For the No-PTSD CG, after assigning randomised index dates between 2012 and 2014, a total of 4,619,992 individuals fulfilled the criterion for being fully observable 2 years prior until 2 years after the assigned random index year or until their death. Of these, 3,123,575 were aged 18–65, and 3,059,008 remained after exclusion of individuals with an F0, F2, or F7 diagnosis 2 years prior to or in the quarter of their index date. After a 1:4 matching by gender and age, a final sample of 51,548 individuals in the No-PTSD CG remained for further analysis. The flowchart in Fig. 1 illustrates the identification process for both groups.

Table 1 displays the sociodemographic characteristics, CCIs, and comparisons between both groups, respectively. In both groups, persons are aged 42.4 years on average (*SD* = 11.8) and 73.4% are female. The PTSD-group includes fewer individuals ‘with dependent coverage’ over family members (15.0%; No-PTSD CG: 19.2%) but more individuals with insurance status ‘retired’ (11.1%; No-PTSD CG: 5.0%). Average CCIs are higher in the PTSD-group (0.87; No-PTSD CG: 0.56). *χ*^2^-tests for insurance status ‘with dependent coverage’ and ‘retired’ as well as the *t* test for CCI show significant differences, with small effect sizes for insurance status ‘retired’ (*V* = 0.10) and CCI (*d* = 0.28, CI 0.26–0.30).

**Table 1:** Sociodemographic characteristics and CCIs for the PTSD (*N* = 12,887) and the No-PTSD CG (*N* = 51,548)

	PTSD	No-PTSD CG	Analysis			
			*χ*^2^	*p*	*V*	
Gender, *n* (%)			0.00	1.000	0.00	
Female	9457 (73.4)	37,828 (73.4)				
Male	3430 (26.6)	13,720 (26.6)				
Insurance status, *n* (%)						
Member	10,296 (79.9)	40,936 (79.4)	1.44	0.231	0.00	
With dependent coverage	1926 (15.0)	9913 (19.2)	125.95	0.000	0.04	
Retired	1428 (11.1)	2562 (5.0)	661.70	0.000	0.10	
			*t*	*p*	*d*	CI_*d*_
Age, mean (SD)	42.37 (11.8)	42.37 (11.8)	0.00	1.000	0.00	0.00–0.00
CCI, mean (SD)	0.87 (1.4)	0.56 (1.1)	− 23.84	0.000	0.28	0.26–0.30

*CCI* Charlson comorbidity index, *n* sample size, *χ*^2^ Chi^2^-test statistic, *tt* test statistic, *p* significance value, *V* Cramer’s *V, d* Cohen’s *d*, *CI* 95% confidence interval

Table 2 shows the overall comorbidity rates separately for all ICD-10-GM chapters and odds ratios. Individuals with PTSD show, nearly overall, higher rates of comorbidities and odds ratios higher than one, especially for mental and behavioural disorders—83% are affected by another mental disorder than PTSD, twice as much as for the No-PTSD CG (41%), with an odds ratio of 7.1. The PTSD-group shows odds ratios above two for chapters VI, XIII, XVIII, XX, and XXII.

**Table 2:** Comorbidities by ICD-10-GM chapters for the PTSD (*N* = 12,887) and the No-PTSD CG (*N* = 51,548)

Chapter	ICD-10-GM	PTSD		No-PTSD CG		Odds ratio
	Description	Persons affected	(%)	Persons affected	(%)	
I	Certain infectious and parasitic diseases	6700	(52.0)	20,086	(39.0)	1.7
II	Neoplasms	4610	(35.8)	16,498	(32.0)	1.2
III	Diseases of the blood and blood-forming organs and certain disorders involving the immune mechanism	1912	(14.8)	5604	(10.9)	1.4
IV	Endocrine, nutritional and metabolic diseases	7162	(55.6)	23,620	(45.8)	1.5
V	Mental and behavioral disorders	10,725	(83.2)	21,220	(41.2)	7.1
VI	Diseases of the nervous system	5784	(44.9)	12,735	(24.7)	2.5
VII	Diseases of the eye and adnexa	5278	(41.0)	18,168	(35.2)	1.3
VIII	Diseases of the ear and mastoid process	3948	(30.6)	11,053	(21.4)	1.6
IX	Diseases of the circulatory system	6565	(50.9)	20,996	(40.7)	1.5
X	Diseases of the respiratory system	9829	(76.3)	33,585	(65.2)	1.7
XI	Diseases of the digestive system	6986	(54.2)	19,422	(37.7)	2.0
XII	Diseases of the skin and subcutaneous tissue	6289	(48.8)	20,830	(40.4)	1.4
XIII	Diseases of the musculoskeletal system and connective tissue	10,236	(79.4)	33,834	(65.6)	2.0
XIV	Diseases of the genitourinary system	8784	(68.2)	32,370	(62.8)	1.3
XV	Pregnancy, childbirth and the puerperium	818	(6.3)	3461	(6.7)	0.9
XVI	Certain conditions originating in the perinatal period	54	(0.4)	292	(0.6)	0.7
XVII	Congenital malformations, deformations and chromosomal abnormalities	2581	(20.0)	7847	(15.2)	1.4
XVIII	Symptoms, signs and abnormal clinical and laboratory findings, not elsewhere classified	9944	(77.2)	29,876	(58.0)	2.5
XIX	Injury, poisoning and certain other consequences of external causes	6974	(54.1)	19,579	(38.0)	1.9
XX	External causes of morbidity and mortality	247	(1.9)	295	(0.6)	3.4
XXI	Factors influencing health status and contact with health services	11,007	(85.4)	41,094	(79.7)	1.5
XXII	Key numbers for specific purposes	121	(0.9)	151	(0.3)	3.2

### Total health care costs

**Fig. 2:**
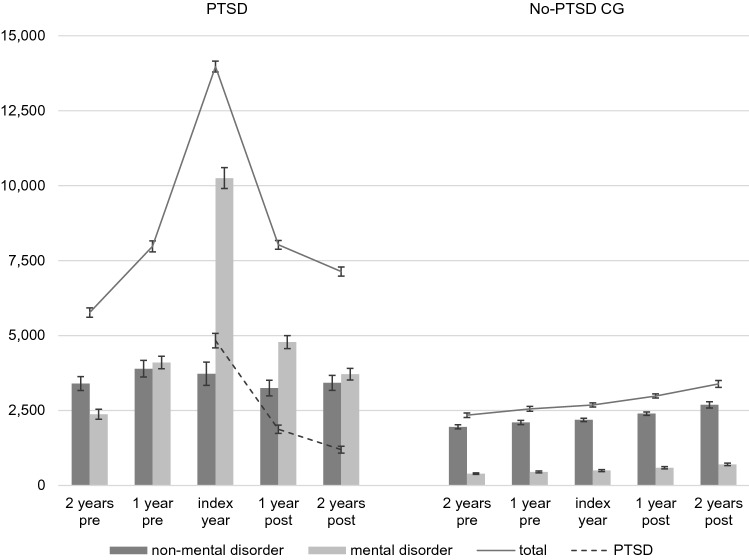
Graphical illustration of changes in total, mental disorder-, non-mental disorder-, and PTSD-specific costs over time for the PTSD (*N* = 12,887) and the No-PTSD CG (*N* = 51,548). Error bars display 95% confidence intervals (CI). Costs are expressed in euros (EUR)

Table 3 shows the total direct and indirect health care costs that arise for both the PTSD and the No-PTSD CG for all analysed outcomes, respectively. In addition, Fig. 2 provides a graphical illustration of changes in total costs as well as in costs due to mental disorders, non-mental disorders and PTSD over the whole course of the analysis period for both groups. For the PTSD-group, costs show a steep upward trend from 2 years prior until the year of index diagnosis, and a corresponding downward trend until 2 years after the index year. Mean costs increase by 142% until the index year (2 years pre 5767 EUR, CI 5537–5996; index year 13,970 EUR, CI 13,585–14,356) and decrease until 2 years after by 49% (7133 EUR, CI 6882–7384). Two years afterwards, costs remain 24% higher than for 2 years preceding the index year. Mean costs 1 year prior (7974 EUR, CI 7696–8251) and 1 year after index year (8026 EUR, CI 7764–8289) are nearly equal. The same trend in mean costs can be seen for all respective outcome variables, except drug costs. Here, mean as well as median costs increase nearly continuously over the analysis period. For total and outpatient treatment costs, medians follow the same trend as the respective mean values; for inpatient treatments, sickness benefit payments, and losses of gross value, all yearly medians remain zero. Total costs for the PTSD-group account for 42,870 EUR (CI 41,909–43,831) over the whole analysis period.

**Table 3:** Total costs resulting for the PTSD (*N* = 12,887) and the No-PTSD CG (*N* = 51,548)

	PTSD				No-PTSD CG				Analysis		
	Median	Mean	SD	CI_mean_	Median	Mean	SD	CI_mean_	*p*	*d*	CI_d_
Drug therapies											
2 years pre	75	447	4069	377–518	31	267	5900	216–318		0.03	0.01–0.05
1 year pre	95	511	4217	439–584	32	290	6303	235–344	*	0.04	0.02–0.06
Index year	129	640	4460	563–717	34	305	2933	279–330	*	0.10	0.08–0.12
1 year post	116	689	5080	602–777	36	336	2279	317–356	*	0.12	0.10–0.14
2 years post	113	724	5199	635–814	37	410	6026	358–462	*	0.05	0.03–0.07
Total	652	3013	21,324	2644–3381	219	1607	20,853	1427–1787	*	0.07	0.05–0.09
Outpatient treatments											
2 years pre	518	808	989	790–825	279	440	572	436–445	*	0.54	0.52–0.56
1 year pre	673	994	1017	976–1011	298	471	618	466–476	*	0.73	0.71–0.75
Index year	1,311	1657	1349	1634–1680	311	495	651	489–500	*	1.39	1.37–1.41
1 year post	989	1398	1370	1374–1422	329	524	688	519–530	*	1.01	0.99–1.03
2 years post	842	1234	1286	1212–1256	348	558	756	551–564	*	0.76	0.74–0.78
Total	5123	6090	4272	6016–6164	1864	2489	2387	2468–2509	*	1.26	1.24–1.28
Inpatient treatments											
2 years pre	0	1292	6202	1185–1399	0	381	1959	364–398	*	0.28	0.26–0.30
1 year pre	0	1783	6245	1675–1891	0	433	2752	409–456	*	0.36	0.34–0.38
Index year	0	3376	8001	3238–3515	0	445	3305	417–474	*	0.63	0.61–0.65
1 year post	0	1749	5817	1649–1850	0	527	2948	501–552	*	0.33	0.31–0.35
2 years post	0	1589	5606	1492–1686	0	563	5358	517–609	*	0.19	0.17–0.21
Total	3275	9790	19,530	9453–10,127	0	2349	9485	2267–2431	*	0.61	0.59–0.63
Sickness benefit payments											
2 years pre	0	265	1581	238–292	0	73	776	67–80	*	0.19	0.17–0.21
1 year pre	0	471	2168	433–508	0	81	838	74–89	*	0.32	0.30–0.34
Index year	0	1163	3584	1101–1225	0	93	899	85–100	*	0.60	0.58–0.62
1 year post	0	451	2066	415–486	0	112	1076	103–121	*	0.25	0.24–0.27
2 years post	0	331	1933	297–364	0	132	1154	122–142	*	0.15	0.13–0.17
Total	0	2681	6769	2564–2797	0	492	2735	468–515	*	0.56	0.54–0.58
Losses of gross value											
2 years pre	0	2955	8314	2812–3099	0	1180	4237	1143–1216	*	0.33	0.32–0.35
1 year pre	0	4215	10,692	4030–4399	0	1278	4456	1239–1316	*	0.47	0.45–0.49
Index year	0	7133	15,250	6870–7397	0	1346	4656	1306–1386	*	0.72	0.70–0.74
1 year post	0	3739	9979	3567–3911	0	1482	5093	1438–1526	*	0.35	0.34–0.37
2 years post	0	3255	9212	3096–3414	0	1721	5768	1671–1771	*	0.23	0.21–0.25
Total	579	21,297	36,432	20,668–21,926	165	7006	16,320	6865–7147	*	0.65	0.63–0.67
Total costs											
2 years pre	1626	5767	13,279	5537–5996	571	2341	8602	2267–2416	*	0.35	0.33–0.37
1 year pre	2346	7974	16,070	7696–8251	617	2552	9209	2473–2632	*	0.50	0.48–0.52
Index year	4501	13,970	22,332	13,585–14,356	650	2683	7868	2615–2751	*	0.92	0.90–0.94
1 year post	2861	8026	15,208	7764–8289	706	2982	8015	2912–3051	*	0.51	0.49–0.53
2 years post	2489	7133	14,539	6882–7384	764	3384	13,193	3270–3498	*	0.28	0.26–0.30
Total	20,801	42,870	55,654	41,909–43,831	5,928	13,942	34,871	13,641–14,243	*	0.73	0.71–0.75

Costs are expressed in euros (EUR)

*CI* 95% confidence intervals

^*^Significant test results for Student’s *t* test after Bonferroni-correction (*p* < 0.001/36 = 0.000027)

In contrast, the No-PTSD CG shows a continuous slight upward trend in costs over the course of the study period. Mean costs increase constantly and in every analysed outcome variable for each of the subsequent years. For mean total costs, an increased rate of 45% can be estimated from 2 years prior to 2 years after the index year (2 years pre: 2341 EUR, CI 2267–2416; 2 years post: 3384 EUR, CI 3270–3498). Median costs for drug therapies, outpatient treatments, and total costs follow the same trend; medians for the other outcome variables remain zero for all years. In total, the No-PTSD CG causes costs of 13,942 EUR (CI 13,641–14,243) per individual over the entire observation period.

Comparisons between groups show significant differences after Bonferroni correction for nearly all outcomes and years. In total, costs for individuals with PTSD are more than three times higher over the total 5-year period compared to those for the No-PTSD CG. A Cohen’s *d* of 0.73 (CI 0.71–0.75) shows an upper mediate effect size. Differences are highest in the index year where the PTSD-group incurs costs five times higher than the No-PTSD CG (means 13,970 EUR, CI 13,585–14,356 versus 2683 EUR, CI 2615–2751), with a large effect size of 0.92 (CI 0.90–0.94). For the years preceding and following the index year, costs are consistently more than twice as high for individuals with PTSD in relation to the non-exposed controls (means 5767–8026 EUR versus 2341–3384 EUR), with small effect sizes for the 2 years preceding (*d* = 0.35; CI 0.33–0.37) as well as the 2 years following (*d* = 0.28; CI 0.26–0.30), and medium effect sizes for 1 year preceding (*d* = 0.50; CI 0.48–0.52) and following (*d* = 0.51; CI 0.49–0.53) the index year. The same results can be seen for all respective outcomes, with the largest effect sizes for outpatient treatments. Only drug therapies show not even small effect sizes, such that differences here can be gauged as very small.

### Health care costs due to mental disorders

Table 4 displays the results for the analysis of costs that lead back to mental disorders only. For the PTSD-group, mental disorder-specific costs show the same trend as total costs, only with higher increase and decrease rates. Mean costs rise by 332% from 2 years preceding (2374 EUR, CI 2208–2540) until the index year (10,251 EUR, CI 9905–10,597) and decrease by 64% until 2 years after the index year (3709 EUR, CI 3517–3901). Costs remain 56% higher 2 years after compared to 2 years preceding the index year. All outcome variables follow the same trend. The highest increase rate occurs in sickness benefit payments; from 2 years prior until the index year, costs increase by 567% (mean pre 144 EUR, CI 123–164; index year: 960 EUR, CI 903–1017). Proportionally to total costs, mental disorder-specific total costs (mean 25,215 EUR, CI 24,501–25,929) account for 59%.

**Table 4:** Mental disorder-specific costs for the PTSD (*N* = 12,887) and the No-PTSD CG (*N* = 51,548)

	PTSD				No-PTSD CG				Analysis		
	Median	Mean	SD	CI_mean_	Median	Mean	SD	CI_mean_	*p*	*d*	CI_d_
Drug therapies											
2 years pre	0	57	243	53–62	0	9	86	9–10	*	0.36	0.34–0.38
1 year pre	0	71	244	67–75	0	10	82	9–10	*	0.47	0.45–0.49
Index year	10	119	315	114–125	0	10	82	9–11	*	0.69	0.67–0.71
1 year post	0	105	309	100–110	0	10	79	10–11	*	0.61	0.59–0.63
2 years post	0	95	279	90–100	0	11	78	10–11	*	0.59	0.57–0.61
Total	51	447	1154	427–467	0	50	348	47–53	*	0.66	0.64–0.68
Outpatient treatments											
2 years pre	97	354	751	341–367	0	91	321	89–94	*	0.59	0.57–0.61
1 year pre	205	501	790	487–515	0	101	348	98–104	*	0.85	0.83–0.87
Index year	767	1164	1115	1145–1184	0	109	367	106–112	*	1.77	1.75–1.79
1 year post	449	912	1179	892–932	0	117	377	114–120	*	1.27	1.25–1.29
2 years post	335	739	1074	720–757	0	128	408	124–131	*	1.01	0.99–1.03
Total	2682	3670	3522	3609–3730	67	546	1382	534–558	*	1.56	1.54–1.58
Inpatient treatments											
2 years pre	0	655	5504	560–750	0	55	853	47–62	*	0.23	0.21–0.25
1 year pre	0	983	4353	908–1058	0	76	1537	63–89	*	0.38	0.36–0.40
Index year	0	2727	7282	2601–2853	0	78	1016	69–87	*	0.78	0.76–0.80
1 year post	0	1188	5193	1098–1278	0	109	1780	93–124	*	0.38	0.37–0.40
2 years post	0	1009	4860	925–1093	0	114	1,635	100–128	*	0.34	0.32–0.36
Total	0	6562	16,712	6274–6851	0	431	3737	399–463	*	0.75	0.73–0.77
Sickness benefit payments											
2 years pre	0	144	1188	123–164	0	26	494	22–31	*	0.17	0.15–0.19
1 year pre	0	311	1774	280–341	0	30	546	25–35	*	0.30	0.28–0.32
Index year	0	960	3297	903–1017	0	34	590	29–39	*	0.59	0.57–0.61
1 year post	0	355	1850	323–387	0	45	770	38–52	*	0.29	0.27–0.31
2 years post	0	242	1706	213–272	0	54	781	48–61	*	0.18	0.16–0.20
Total	0	2011	5796	1911–2111	0	190	1773	175–206	*	0.60	0.58–0.62
Losses of gross value											
2 years pre	0	1165	5988	1062–1268	0	210	2326	190–230	*	0.28	0.26–0.30
1 year pre	0	2233	8627	2084–2382	0	234	2494	213–256	*	0.45	0.43–0.47
Index year	0	5281	14,021	5039–5523	0	265	2736	242–289	*	0.75	0.73–0.77
1 year post	0	2222	8703	2071–2372	0	306	3068	279–332	*	0.40	0.38–0.42
2 years post	0	1624	7531	1494–1754	0	392	3497	361–422	*	0.27	0.25–0.29
Total	0	12,524	28,597	12,031–13,018	0	1,407	8479	1334–1480	*	0.75	0.73–0.77
Total costs											
2 years pre	127	2374	9620	2208–2540	0	392	3119	365–419	*	0.39	0.37–0.41
1 year pre	317	4099	12,064	3890–4307	0	451	3577	420–481	*	0.58	0.56–0.60
Index year	2029	10,251	20,057	9905–10,597	0	496	3722	464–528	*	1.02	1.00–1.04
1 year post	760	4782	12,502	4566–4998	0	587	4447	549–625	*	0.61	0.59–0.63
2 years post	509	3709	11,105	3517–3901	0	698	4850	657–740	*	0.46	0.44–0.48
Total	7505	25,215	41,341	24,501–25,929	82	2624	11,916	2521–2727	*	1.06	1.04–1.08

Costs are expressed in euros (EUR)

*CI* 95% confidence intervals

^*^Significant test results for Student’s *t* test after Bonferroni-correction (*p* < 0.001/36 = 0.000027)

The same trend as for total costs can also be seen for the No-PTSD CG. In all outcome variables, costs increase constantly in every year. Total costs due to mental disorders increase by 78% from 2 years preceding (mean 392 EUR, CI 365–419) until 2 years after the index year (mean 698 EUR, CI 657–740). Average mental disorder-specific costs of 2624 EUR (CI 2521–2727) in total per individual account for 19% of all occurring health care costs which accumulated over the whole analysis period.

All *t* tests that were conducted between the PTSD and the No-PTSD CG show significant results. Compared to total costs, differences show even higher discrepancies for mental disorder-specific health care costs. In total, the PTSD-group causes costs 9.6 times higher as compared to the No-PTSD CG, with a large effect size (*d* = 1.06; CI 1.04–1.08). In the year of index diagnosis, costs due to mental disorders are 20.7 times higher in the PTSD-group (means 10,251 EUR, CI 9905–10,597 versus 496 EUR, CI 464–528), with a large effect size of *d* = 1.02 (CI 1.00–1.04). For all other analysed periods, yearly total costs are between five to nine times higher in the PTSD-group (means 2374–4782 EUR versus 392–698 EUR), with small to medium effect sizes (*d* = 0.39–0.61). The same findings can be seen for all respective outcome variables, with the largest effect sizes for outpatient treatments (*d* = 0.59–1.77).

### Health care costs not due to mental disorders

In Table 5, results for analyses of costs not due to mental disorders are shown. In contrast to trends for total and mental disorder-specific analyses, results in the PTSD-group show different trends for non-mental disorder costs. Total health care costs remain stable from 2 years preceding (mean 3396 EUR, CI 3243–3550) until 2 years after the index year (3419 EUR, CI 3265–3573). Total health care costs show their maximum at 1 year preceding the index year (mean 3892 EUR, CI 3706–4078) and decrease slightly in the year of index diagnosis (mean 3725 EUR, CI 3543–3907), which is 10% higher compared to 2 years prior. Trends for outcome variables differ slightly: costs for drug therapies increase constantly every year, in total by 61% (mean 2 years pre 390 EUR, CI 320–460; 2 years post 629 EUR, CI 540–719). Costs for outpatient treatments increase as well, but only by 9% over the whole period (mean 2 years pre 454 EUR, CI 443–464; 2 years post: 495 EUR, CI 484–506). For inpatient treatments, sickness benefit payments, and losses of gross value, costs not due to mental disorders two years after the index year are lower compared to 2 years prior (decrease rates: 10%, 36%, 10%). Most confidence intervals for mean costs show overlaps in each respective outcome variable. Total health care costs not due to mental disorders of 17,677 EUR (CI 17,087–18,267) on average account for 41% of total health care costs in the PTSD-group.

**Table 5:** Non-mental disorder-specific costs for the PTSD (*N* = 12,887) and the No-PTSD CG (*N* = 51,548)

	PTSD				No-PTSD CG				Analysis		
	Median	Mean	SD	CI_mean_	Median	Mean	SD	CI_mean_	*p*	*d*	CI_*d*_
Drug therapies											
2 years pre	56	390	4057	320–460	29	257	5898	207–308		0.02	0.00–0.04
1 year pre	65	441	4204	368–513	30	280	6301	226–334		0.03	0.01–0.05
Index year	70	521	4441	444–598	32	295	2929	269–320	*	0.07	0.05–0.09
1 year post	72	584	5063	497–672	33	326	2275	306–346	*	0.09	0.07–0.10
2 years post	72	629	5181	540–719	34	399	6025	347–451	*	0.04	0.02–0.06
Total	405	2565	21,255	2198–2932	205	1557	20,843	1377–1737	*	0.05	0.03–0.07
Outpatient treatments											
2 years pre	302	454	603	443–464	231	349	445	345–353	*	0.22	0.20–0.24
1 year pre	332	493	604	482–503	246	370	482	366–374	*	0.24	0.22–0.26
Index year	310	493	717	480–505	254	386	508	382–390	*	0.19	0.17–0.21
1 year post	307	486	640	475–497	266	407	542	403–412	*	0.14	0.12–0.16
2 years post	317	495	641	484–506	279	430	600	425–435	*	0.11	0.09–0.13
Total	1876	2421	2107	2384–2457	1533	1942	1762	1927–1958	*	0.26	0.24–0.28
Inpatient treatments											
2 years pre	0	637	2650	591–682	0	326	1682	312–341	*	0.16	0.14–0.18
1 year pre	0	804	4530	726–882	0	357	2042	339–374	*	0.16	0.15–0.18
Index year	0	648	3018	596–700	0	367	3076	341–394	*	0.09	0.07–0.11
1 year post	0	560	2215	521–598	0	418	2192	399–437	*	0.06	0.05–0.08
2 years post	0	579	2347	539–620	0	450	5009	406–493	*	0.03	0.01–0.05
Total	295	3227	8156	3087–3368	0	1918	7910	1849–1986	*	0.17	0.15–0.18
Sickness benefit payments											
2 years pre	0	121	1054	103–140	0	47	599	42–52	*	0.10	0.09–0.12
1 year pre	0	162	1279	140–184	0	51	634	46–57	*	0.14	0.12–0.16
Index year	0	205	1522	178–231	0	58	676	52–64	*	0.16	0.14–0.18
1 year post	0	96	937	80–112	0	67	754	60–73		0.04	0.02–0.06
2 years post	0	89	915	73–104	0	78	848	71–85		0.01	− 0.01 to 0.03
Total	0	672	3262	616–729	0	301	1995	284–319	*	0.16	0.14–0.18
Losses of gross value											
2 years pre	0	1794	5742	1695–1893	0	971	3489	941–1001	*	0.20	0.18–0.22
1 year pre	0	1993	6424	1882–2104	0	1043	3635	1011–1074	*	0.22	0.20–0.24
Index year	0	1858	6636	1744–1973	0	1080	3691	1048–1111	*	0.18	0.16–0.20
1 year post	0	1520	4814	1437–1603	0	1176	4008	1142–1211	*	0.08	0.06–0.10
2 years post	0	1626	5115	1538–1714	0	1329	4506	1290–1368	*	0.06	0.05–0.08
Total	0	8792	18,815	8467–9116	0	5599	12,841	5488–5709	*	0.22	0.21–0.24
Total costs											
2 years pre	851	3396	8913	3243–3550	478	1951	7915	1883–2019	*	0.18	0.16–0.20
1 year pre	954	3892	10,774	3706–4078	507	2101	8265	2030–2172	*	0.20	0.18–0.22
Index year	849	3725	10,540	3543–3907	534	2186	6726	2127–2244	*	0.20	0.18–0.22
1 year post	923	3245	8450	3100–3391	568	2394	6390	2339–2450	*	0.12	0.11–0.14
2 years post	954	3419	8918	3265–3573	617	2685	12,067	2581–2790	*	0.06	0.04–0.08
Total	7894	17,677	34,196	17,087–18,267	5024	11,317	31,521	11,045–11,590	*	0.20	0.18–0.22

Costs are expressed in euros (EUR)

*CI* 95% confidence intervals

^*^Significant test results for Student’s *t* test after Bonferroni-correction (*p* < 0.001/36 = 0.000027)

Again, the same trends can be seen in all outcome variables for the No-PTSD CG with respect to total and mental disorder-specific costs. Mean costs increase constantly in every year, in total by 38%, from 1951 EUR (CI 1883–2019) 2 years prior to 2685 EUR (CI 2581–2790) 2 years after the index year. In total, mean costs not due to mental disorders accumulate to 11,317 EUR (CI 11,045–11,590), which accounts for 81% of total health care costs over the whole analysis period.

Comparisons between groups show significant differences for nearly all outcomes and periods and the PTSD-group causes higher costs for every year as well. In any event, differences are far lower compared to total and mental disorder-specific costs. Ratios for total costs range only from 1.3 to 1.9 per year, and with very up to slightly small effect sizes (*d* = 0.06–0.26). In total, the PTSD-group incurs non-mental disorder-specific costs that are 1.6 times higher.

### Health care costs due to PTSD

For broader clarity in estimating costs that arise due to PTSD, Table 6 displays the expenses that are caused specifically by PTSD after index diagnosis in the PTSD-group as well as the number of patients treated. Costs are highest in the index year for all outcome variables and consistently decrease in both the following years. One year after the index year, PTSD-specific total costs decrease by 61% (4830 EUR, CI 4590–5070 versus 1873 EUR, CI 1734–2012); another year later, costs reduce again by 36% (1192 EUR, CI 1077–1307). In total, PTSD-specific costs decrease by 75%. Reduction rates for single outcome variables range from 56 to 80%. The amount for the entire period (mean 7895 EUR, CI 7523–8266) accounts for 18% of the total direct and indirect costs for individuals with PTSD (mean 42,870 EUR), proportionally in the year of index diagnosis for 35% (4830 EUR of 13,970 EUR). In the index year, all individuals with PTSD utilise PTSD-specific health care services; 1 year after, only 7110 (55%) do so, and 2 years later only 5827 (45%). The same trends can be observed for all respective outcomes.

**Table 6:** PTSD-specific costs for the PTSD-group (*N* = 12,887)

	Patients treated	(%)	Median	Mean	SD	CI_mean_
Outpatient treatments						
Index year	12,116	(94.0)	252	603	838	588–617
1 year post	6971	(54.0)	59	385	797	371–398
2 years post	5730	(44.5)	0	266	627	255–276
Total	12,245	(95.0)	498	1253	1901	1220–1286
Inpatient treatments						
Index year	2043	(15.8)	0	1627	5728	1529–1726
1 year post	529	(4.1)	0	426	2938	375–477
2 years post	436	(3.4)	0	330	2822	281–379
Total	2387	(18.5)	0	2384	8442	2238–2529
Sickness benefit payments						
Index year	696	(5.4)	0	405	2253	366–444
1 year post	362	(2.8)	0	156	1236	135–177
2 years post	157	(1.2)	0	78	972	62–95
Total	878	(6.8)	0	639	3250	583–695
Losses of gross value						
Index year	1280	(9.9)	0	2195	9608	2029–2361
1 year post	579	(4.5)	0	906	5781	806–1006
2 years post	350	(2.7)	0	518	4627	438–598
Total	1601	(12.4)	0	3619	15,139	3357–3880
Total costs						
Index year	12,887	(100.0)	425	4830	13,917	4590–5070
1 year post	7110	(55.2)	70	1873	8035	1734–2012
2 years post	5827	(45.2)	0	1192	6665	1077–1307
Total	12,887	(100.0)	944	7895	21,516	7523–8266

Costs are expressed in euros (EUR)

*CI* 95% confidence intervals

## Discussion

### Interpretation of results

In this study, we compared changes in health care costs over time and determined estimates for incremental costs that arise for individuals with an incident diagnosis of PTSD, in relation to an age and gender-adjusted control group of non-exposed individuals. The findings suggest that overall health care costs for individuals with PTSD were more than three times higher compared to non-exposed controls in a 5-year period (42,870 EUR versus 13,942 EUR). Costs for the PTSD-group are at least twice as high in every year preceding and following index diagnosis. Hence, individuals with PTSD seem to suffer from more complex disorder conditions and more severe preceding and continuing overall health impairments.

Most of these impairments and incremental costs seem to be caused by PTSD itself and co-occurring mental disorders. This is supported by the analyses on costs and cost developments as well as by the results for co-occurring disorders. CCIs are slightly higher and co-occurrence rates are nearly overall increased in the PTSD-group but are the highest by far for mental and behavioural disorders, with an odds ratio of 7.1. In terms of costs, mental disorder-specific health care costs account for 59% of overall costs in the PTSD-group, but only for 19% in the No-PTSD CG. In the No-PTSD CG, costs increase constantly year by year at a small rate, which can be explained by overall increases in health care expenditures within the German statutory health care system [32]. Absolute mean costs for non-mental disorder-specific utilisations are roughly 60% higher in total in the PTSD-group but remain nearly stable over the whole analysis period. In contrast, total costs and costs due to mental disorders arise drastically in the index year of a PTSD diagnosis but return to their initial level afterwards.

Overall, individuals with PTSD cause around 29,000 EUR more in costs over a 5-year period compared to non-exposed average insurants. Of these, incremental costs specifically due to PTSD only can be estimated at roughly 8000 EUR, equal to 27% of total incremental costs; 73% of these costs seem to be due to other disorders, mainly other mental disorders. However, total costs show a proportionally lower increase rate from 2 years preceding the index year until 2 years after for individuals with PTSD compared to the No-PTSD CG (23% versus 45%). Taking these trends into account, we could assume that treatments covered in the statutory health care system lead to a decline of symptom severity and could counterbalance an outbreak of PTSD in the long run. International guidelines highly recommend psychotherapy with additional pharmacotherapy if necessary as the treatment option of choice for PTSD [33, 34] and empirical evidence supports their effectiveness [35]. 94% of individuals with PTSD utilised PTSD-specific outpatient services in the year after index diagnosis. Despite no clear conclusions on these terms can be drawn, the results let assume that focussing treatments for mental health impairments, e.g. psychotherapies or pharmacotherapies, are beneficial for individuals with PTSD and early preventive approaches could prevent high incremental costs.

Previous research on costs due to mental disorders shows different results, along with a lack of standardised procedures. Due to different methodological approaches, comparability with other research on costs stemming from PTSD and other mental disorders is restricted. Compared to one US-American study on Medicaid and private insurance data, our results correspond with the findings. The study estimated direct costs due to PTSD at 10,960–18,753 USD per patient per year [23], which is comparable to the reported direct and indirect costs of 13,970 EUR in the year of index diagnosis found in this study. In contrast, a Northern Ireland study estimated annual costs of 4236 EUR for individuals with 12-month prevalent PTSD [24], which is remarkably lower. Compared to research on costs due to other mental disorders, our results seem to be in an expected range. Research for borderline personality disorders (BPD, ICD-10-GM F60.3) estimated yearly costs of 16,852 EUR per affected individual [36], which is comparable to our findings. A systematic review on costs due to eating disorders reported annual costs per patient of 1288–8042 USD [17], which again is lower than our findings. A similar cost-analysis on claims data with comparable estimation approaches found costs of 8290 EUR for an incident BPD and 3616 EUR for an incident major depressive disorder (ICD-10-GM F32 and F33) in the year of index diagnosis [37].

Compared to these findings, costs due to PTSD turn out to be higher than for other mental disorders. Considering the complexity and severity of PTSD and high co-occurrence rates for mental and physical disorders, high costs for PTSD seem reasonable. Due to the large sample size and conservative estimation approaches, our results can be considered good estimates for costs due to PTSD. Moreover, this study describes cost developments over a long analysis period preceding and following incident PTSD. In addition, the results add evidence as to the high financial and economic burden that PTSD causes for a welfare state, along with the immersive health restrictions for affected individuals.

### Limitations

Several limitations should be considered for this study. First, studies on claims data are generally associated with restrictions in data quality compared to the quality of primary data. Especially relevant for this study is the fact that no statements on the validity of diagnoses can be made. We tried to face these issues by defining strict inclusion and exclusion criteria; e.g., only secure diagnoses by inpatient services, or two or more outpatient diagnoses or one by a mental disorder specialist. Furthermore, due to data constraints, we could only include part of the cost factors that might be relevant. Because information is only available from the perspective of health insurers, data for other direct and indirect expenses—e.g., out-of-pocket expenses, early retirements, life quality, and thus, quality-adjusted life years—as well as reliable information on disease remissions for assessing whether or not treatments were successful could not be included. Additionally, data for short-term incapacities to work are probably not covered by claims data [29] so that results for this outcome are probably underestimated. Due to insufficient data on diagnosis-specific medications, drug-therapy costs could not be included in the analysis of costs due to PTSD-specific services. Thus, expressed costs are only estimates, and real occurring costs in total as well as PTSD-specific costs are probably even higher. At any rate, the overall quality of German claims data can be rated as good [38] and, considering the available data sources and the sample sizes, our findings should suffice as good estimates. As only scant information on the amount of incremental costs due to PTSD is available, our study contributes to research on this disease and on mental disorders overall.

Differences in sociodemographic data could also restrict comparability between groups; one could also argue that we should have expanded our matching procedure to characteristics beyond gender and age, or applied Propensity Score Matchings [39]. Considering the explorative approach of this study and the aim of identifying estimates for incremental costs due to PTSD compared to non-diseased average insurants, we consciously refrained from using broader approaches. Controlling only by gender and age enables comparisons between individuals who suffer from PTSD and non-exposed average controls so that conclusions about the overall health status and costs arising from PTSD in relation to average insurants can be drawn. Furthermore, the chosen procedure enables estimations of costs due to symptoms and co-occurring disorders before an index diagnosis of PTSD; controlling for, e.g., CCIs or co-occurrence rates would have inhibited this.

## Conclusions

This study provides estimates for incremental costs due to incident PTSD and describes trends of cost developments arising for the health care system and welfare state over a 5-year period. Individuals with PTSD cause costs of approximately 43,000 EUR in total, three times higher than for non-exposed average insurants. All in all, incremental costs can be estimated at roughly 29,000 EUR and lead back mostly to PTSD and to other co-occurring mental and behavioural disorders. Furthermore, the findings support evidence on the complexity and severity of health restrictions caused by PTSD due to co-occurring disorders, especially accompanying mental disorders, which account for double the costs in relation to PTSD-specific costs only. Taking the overall reduction rates in cost developments after an onset of PTSD into account, this study suggests that treatments for PTSD are efficacious and benefit affected individuals. Thus, health policies should focus overall on enhancing and encouraging early evidence-based treatments and prevention approaches. Further research should investigate precise cost–benefit estimates for treatment approaches for PTSD and other mental disorders in general on claims data.
